# Cost of illness of hyponatremia in the United States

**DOI:** 10.1186/1478-7547-4-10

**Published:** 2006-05-31

**Authors:** Audra Boscoe, Clark Paramore, Joseph G Verbalis

**Affiliations:** 1 United BioSource Corporation, Bethesda, MD, USA; 2 Georgetown University Medical Center, Georgetown University, Washington, DC, USA

## Abstract

**Background:**

Hyponatremia is a disorder of fluid and electrolyte balance characterized by a relative excess of body water relative to body sodium content. It is the most common electrolyte disorder encountered in clinical medicine and is associated with negative outcomes in many chronic diseases. However, there is limited information in the literature about health care resource use and costs attributable to the effects of the condition. The purpose of this analysis was to estimate the annual cost of illness of hyponatremia in the United States.

**Methods:**

The study utilized a prevalence-based cost of illness framework that incorporated data from publicly available databases, published literature and a consensus panel of expert physicians. Panel members provided information on: classification of hyponatremia patients, treatment settings for hyponatremia (i.e., hospital, emergency room, doctor's office), and health care resource use associated with the diagnosis and treatment of hyponatremia. Low and high prevalence scenarios were estimated and utilized in a spreadsheet-based cost of illness model. Costs were assigned to units of resources and summarized across treatment settings.

**Results:**

The prevalence estimate for hyponatremia ranged from 3.2 million to 6.1 million persons in the U.S. on an annual basis. Approximately 1% of patients were classified as having acute and symptomatic hyponatremia, 4% acute and asymptomatic, 15%–20% chronic and symptomatic, and 75–80% chronic and asymptomatic. Of patients treated for hyponatremia, 55%–63% are initially treated as inpatients, 25% are initially treated in the emergency room, and 13%–20% are treated solely in the office setting. The direct costs of treating hyponatremia in the U.S. on an annual basis were estimated to range between $1.6 billion and $3.6 billion.

**Conclusion:**

Treatment of hyponatremia represents a significant healthcare burden in the U.S. Newer therapies that may reduce the burden of hyponatremia in the inpatient setting could minimize the costs associated with this condition.

## Background

Hyponatremia, defined as a serum sodium concentration ([Na^+^]) less than 135 mEq/L [1], represents a relative excess of body water relative to body sodium content. Clinical symptoms are largely related to dysfunction of the central nervous system, and are more evident when the decrease in the serum sodium concentration is large or fast [2]. Although most hyponatremic patients may appear to be asymptomatic, severe symptomatic hyponatremia is a medical emergency that calls for immediate treatment. Complications of severe and rapidly developing hyponatremia can include seizures, coma, brain-stem herniation, respiratory arrest, permanent brain damage, and death [3].

Hyponatremia is the most common electrolyte disorder encountered in clinical medicine [3]. Incidence rates as high as 15%–22% have been reported in hospitalized patients in intensive care units [4] or long-term care facilities [5]. However, most studies have reported a hospital-based incidence of 1%–4% for more clinically significant cases of hyponatremia (i.e., serum [Na^+^] less than 130 mEq/L) [3]. There are no published estimates of the prevalence of hyponatremia in the U.S. Miller, Morley, and Rubenstein [6] reviewed medical charts for 119 nursing home patients and found that 53% had at least one episode of hyponatremia over a one-year period. More recently, Hawkins [7] examined the prevalence of hyponatremia in 120,137 patients at initial presentation to healthcare providers in Singapore, and reported a range from 7.2% in the community care setting to as high as 28.2% for acute care hospitalized patients.

Hyponatremia has also been associated with negative outcomes in many chronic diseases, most notably in patients with congestive heart failure [8]. One study of 161 patients with severe congestive heart failure found hyponatremia to be a significant predictor of cardiovascular mortality, with 69% of hyponatremic patients dying within 24 months as compared with 40% of patients without baseline hyponatremia (*P *< 0.001) [9]. Results from a prospective study of 435 hospitalized patients with congestive heart failure indicated that a serum [Na^+^] less than or equal to 135 mEq/L was a significant (*P *< 0.01) and independent predictor of major complication or death during hospitalization; 25% of patients with a serum [Na^+^] less than or equal to 135 mEq/L, versus 15% of those with a serum [Na^+^] greater than 135 mEq/L experienced a major complication or died [10]. Similarly, in a study examining admission hyponatremia among 4,123 geriatric patients, in-hospital mortality was 16% among patients with admission hyponatremia versus 8% among those without this condition [11]. And in a general adult hospitalized population, Anderson et al. [12] found that mortality rates were 60-fold higher in patients with even asymptomatic hyponatremia compared to normonatremic patients. The degree to which this strong association between hyponatremia and negative outcomes is causally related to the hyponatremia, and might be improved with more effective therapies, is not known.

There is limited information in the literature about health care resource use and costs attributable to the effects of hyponatremia. This may be due to the low incidence of clinically significant hyponatremia, or due to methodological challenges of isolating the effects of the condition since morbidity and mortality are often related to the underlying medical disorder. Two studies in patients with congestive heart failure have determined that hyponatremia is a significant predictor of increased length of stay[10,13]. To our knowledge, no studies have been conducted assessing the cost of illness of hyponatremia in different treatment settings. Such information would be useful given the likely variation in intensity of resource use and costs of care associated with hyponatremia.

Against this background, the present study utilized a prevalence-based cost-of-illness framework to estimate the annual cost of illness of hyponatremia in the U.S. The analysis incorporates data from publicly available databases, published literature, and an expert physician panel. The resulting cost of illness estimate is presented from the payor perspective and focuses on direct treatment costs, while excluding indirect costs (i.e., worker productivity losses) that may be associated with hyponatremia.

## Methods

We used a prevalence-based epidemiologic model to estimate the annual direct costs of hyponatremia in the U.S. [14]. A differential approach was used; to focus on the excess burden of hyponatremia, costs related to any diagnosis or underlying disease other than hyponatremia were not taken into account [15]. The two main sources of data for the analysis were the published literature and an expert panel. Indirect costs were not included in the analysis as the expert panel did not feel qualified to assign levels of work loss or caregiver burden based on the presence of hyponatremia.

### Expert panel

Expert opinion was used in this study because neither the published literature nor national surveys or databases contain adequate information on the health care resource use and costs associated with hyponatremia. The role of the expert panel was two-fold: first, to provide a classification scheme for hyponatremia patients, and second, to estimate the health care resource use associated with the diagnosis and treatment of hyponatremia.

Our goal was to choose physicians who are representative of the types of physicians who encounter hyponatremia in practice, and are considered experts in the field [16]. An endocrinologist was chosen as lead physician based on a review of the published hyponatremia literature. The lead physician then provided recommendations for other panel members with an extensive background and experience in treating patients with hyponatremia. The expert panel was comprised of six physicians from different specialties, including two endocrinologists, one nephrologist, one cardiologist, one internist, and one intensivist.

A consensus panel was utilized to estimate desired model parameters for patients with hyponatremia [17]. This approach was utilized by Murray et al. in their study of the cost of refractory epilepsy [18], and by Plumb and Guest in their analysis of the cost of erectile dysfunction in the UK [19]. A detailed questionnaire was mailed to panel members in advance of a face-to-face meeting. The panel members completed the questionnaire prior to the meeting and the responses were summarized and presented to the panel on the day of the meeting. The questionnaire results and other issues were then discussed among the panel members until agreement was reached. Previous research has found that consensus panel decisions have a high degree of consistency and validity when compared to clinical practice [20,21].

The questionnaire covered the following topic areas: classification of hyponatremia patients, health care resource use associated with the diagnosis of hyponatremia, initial treatment settings for hyponatremia, health care resource use associated with the treatment of hyponatremia, and the treatment of hyponatremia-related complications.

### Classifying hyponatremia patients

The first step in establishing the economic burden of a given disease or condition is to characterize the patient population with the condition. The expert panel was asked to provide a classification scheme for hyponatremic patients that correlated well with the levels of health care resource use. For example, if there were two main types of hyponatremic patients, and one type never used health care services while the other type had frequent hospitalizations, this distinction would be critical for an economic evaluation. Four classification options were presented to the panel including: 1) acute [developing within 48 hours] vs. chronic [unknown duration or duration greater than 48 hours] hyponatremia, 2) symptomatic vs. asymptomatic hyponatremia, 3) a combination of the first two options (i.e., acute symptomatic, acute asymptomatic, chronic symptomatic, chronic asymptomatic), or 4) based on underlying condition (e.g., congestive heart failure, syndrome of inappropriate antidiuretic hormone secretion [SIADH]). The panel agreed unanimously to base the economic evaluation on the third option.

The panel was not able to provide a specific percentage breakdown of hyponatremia patients into the four categories, but did provide a range of percentages for each category. For the purposes of estimating the cost of illness of hyponatremia, we utilized an approach similar to the one used by Severens et al in their analysis of the cost of pressure ulcers in the Netherlands whereby the ranges provided by the expert panel were converted into "low" and "high" estimates [22] (described in greater detail below).

### Estimating prevalence of hyponatremia

The ability to estimate the prevalence of hyponatremia in the U.S. population was enabled by the availability of two key data elements. First, publicly available hospital discharge data provided empirical evidence of how many patients are treated for hyponatremia in an inpatient setting each year in the U.S. The U.S. Government's Healthcare Cost & Utilization Project (HCUP) database contains hospital discharge data from a 20% sample of U.S. hospitals (approximately 7 million hospital stay records from 1,000 hospitals in 33 states) and yields nationally representative estimates of inpatient care [23]. In 2002 there were an estimated 923,473 hospital stays with either a principal or secondary discharge diagnosis of hyponatremia (ICD-9-CM diagnosis code 276.1). We assumed an average of 1.25 hospital stays per patient, based on a study by Tierney et al. [24] which reported 954 admissions for the 763 hyponatremic patients in their sample, to arrive at an estimated 738,778 patients treated for hyponatremia in an inpatient setting in the U.S.

Second, using the four-level classification system, the expert panel provided "low" and "high" estimates of the proportion of hyponatremic patients who are treated; and "low" and "high" estimates of the proportion initially treated in an inpatient setting (Table 1). All possible combinations of the three sets of low and high estimates in Table 1 (e.g., one combination would include the "low" estimate for classification, the "low" estimate for percentage treated, and the "low" estimate for percentage treated as inpatient) were evaluated and resulted in eight separate estimates of the proportion of all hyponatremic patients who are treated in an inpatient setting. Based on these calculations, we were able to extrapolate from the number of "known" hospitalized hyponatremia patients (i.e. 738,778 patients) to produce eight separate estimates of the total number of persons with hyponatremia in the U.S.

**Table 1:** Expert panel estimates used in prevalence calculations

**Classification of hyponatremia patients**	**Low**	**High**
Acute and symptomatic	1%	1%
Acute and asymptomatic	4%	4%
Chronic and symptomatic	20%	15%
Chronic and asymptomatic	75%	80%
	100%	100%

**Percent of hyponatremia patients treated**	**Low**	**High**

Acute and symptomatic	90%	100%
Acute and asymptomatic	90%	100%
Chronic and symptomatic	66%	85%
Chronic and asymptomatic	10%	20%

**Of those treated, percent treated initially as inpatient**	**Low**	**High**

Acute and symptomatic	65%	75%
Acute and asymptomatic	65%	75%
Chronic and symptomatic	40%	45%
Chronic and asymptomatic	70%	80%

The following example using the "low" values for each of the three parameters illustrates our methodology for calculating the prevalence estimate. In this scenario, for every 100,000 individuals with hyponatremia, 1,000 (1%) are acute and symptomatic. Of those, 900 are treated (90%), and 585 (65% of those treated) are treated in an inpatient setting. By adding the 585 acute and symptomatic patients to the similarly derived values for the acute asymptomatic, chronic symptomatic, and chronic asymptomatic groups, we determined there were a total of 13,455 patients treated for hyponatremia in an inpatient setting for every 100,000 individuals with hyponatremia. Given that an estimated 738,778 patients were treated in an inpatient setting in 2002, the total number of individuals with hyponatremia in the U.S. using this particular combination of estimates was 5.49 million (738,778 × [100,000/13,455]).

We repeated this procedure for all eight possible combinations of estimates (e.g. "low", "high", "low"; "high", "high", low"). The lowest and highest of the eight resulting prevalence estimates were then used in subsequent cost of illness calculations (i.e., "low" scenario and "high" scenario).

While hyponatremia is defined as a serum sodium concentration ([Na^+^]) less than 135 mEq/L [1], the panel felt that a serum sodium concentration ([Na^+^]) less than 130 mEq/L is the threshold for clinically significant hyponatremia, and therefore the level physicians would consider the threshold for initiating treatment. Accordingly, the expert panel's estimates of treatment patterns, and therefore our estimates of prevalence, were based on a conservative assumption that only patients with clinically significant hyponatremia (serum sodium concentration ([Na^+^]) less than 130 mEq/L) are being treated.

### Estimating health care resource use

To simplify the costing exercise, the panel first reached consensus on a mutually exclusive list of initial treatment settings for patients with hyponatremia, and low and high estimates for the percentage of patients treated in each setting: inpatient, emergency room (ER) (without being hospitalized), or doctor's office (without being hospitalized or visiting the emergency room) (Table 2). For those admitted as an inpatient, the panel estimated the proportion admitted specifically for hyponatremia; and the average, incremental increase in length of stay due to hyponatremia for patients admitted due to other conditions. The panel provided detailed information on the hyponatremia-related tests and procedures that are performed in each treatment setting, and the proportion of patients receiving each test or procedure. This included both diagnostic and therapeutic tests and procedures. The frequency and resource use intensity of follow-up visits were also estimated by the panel. The panel's estimates of the proportion of patients receiving each test and procedure performed at the initial evaluation varied depending on the patient's underlying condition (Table 3). Differences in the tests and procedures performed were based on whether the patient's etiology was: 1) SIADH; 2) congestive heart failure, cirrhosis, renal failure, or diuretics; or 3) any other etiology. The panel provided estimates of the percentage of hyponatremia patients who fell into each of the three etiological categories. These percentages enabled us to calculate an absolute number of patients receiving each test and procedure by underlying condition at initial evaluation. Estimates of the proportion of patients receiving each test and procedure performed at follow-up were based on the initial treatment setting. The number of patients receiving each test and procedure at initial evaluation and follow-up were multiplied by unit prices to determine the contribution of tests and procedures to the total cost of illness.

**Table 2:** Treatment by setting for hyponatremia patients

	**Inpatient**	**ER**	**Office/Clinic**
**Low Scenario (3.16 million prevalence; 1.17 million treated patients)**			
Overall	**63% (738,778)**	**24% (280,988)**	**13% (148,387)**
Acute and Symptomatic	75% (23,679)	25% (7,893)	0% (0)
Acute and Asymptomatic	75% (94,715)	25% (31,572)	0% (0)
Chronic and Symptomatic	45% (241,524)	45% (241,524)	10% (53,672)
Chronic and Asymptomatic	80% (378,861)	0% (0)	20% (94,715)

**High Scenario (6.07 million prevalence; 1.35 million treated patients)**			

Overall	**55% (738,778)**	**25% (333,070)**	**20% (274,036)**
Acute and Symptomatic	65% (35,530)	35% (19,131)	0% (0)
Acute and Asymptomatic	65% (106,590)	35% (57,394)	0% (0)
Chronic and Symptomatic	40% (256,544)	40% (256,544)	20% (128,272)
Chronic and Asymptomatic	70% (340,115)	0% (0)	30% (145,764)

**Table 3:** Unit costs

**Resource Item**	**Office**	**ER/Inpatient**	
	**Professional Fee**	**Professional Fee**	**Facility Fee/Lab Fee**

**Office and ER Costs**			
Initial Visit	$110.50	$130.77	$157.50
Follow-up visit	$71.00	N/A	N/A
Chest X-Ray	$122.00	$42.57	$55.50
MRI	$1,852.50	$388.28	$382.00
Chest CT	$1,067.50	$320.68	$352.00
Abdominal CT	$923.00	$253.94	$281.50
Urine osmolality	$31.00	$31.00	$9.52
TSH	$66.00	$66.00	$23.47
ACTH stimulation test	$159.50	$159.50	$53.97
Basic metabolic panel	$39.50	$39.50	$11.83
Venipuncture			$3.00
**Inpatient costs**			
*Patients admitted for hyponatremia*			
Total cost of hospital stay			$6,926.00
*Patients admitted for another condition*			
Per diem (general ward)			$500.00
Per diem (ICU)			$1,100.00
**Consultation**		$106.00	

Again, the panel provided a range of estimates for many of the resource use items. Therefore, when calculating the cost of illness based on the "low" prevalence scenario, we utilized the low end of the range of resource use estimates from the expert panel, and vice versa for the "high" prevalence scenario. This approach resulted in both the most conservative and most generous cost of illness estimates.

The questionnaire also addressed neurological complications due specifically to hyponatremia. However, the panel agreed that given how infrequently these arise, they could not provide an accurate estimate of the percentage of patients who would incur costs for complication-related resource use. In the rare cases in which a patient does develop complications, costs are substantial; but because the number of patients affected is small and could not be confidently quantified, these costs have not been included in the analysis.

### Cost assignment

The cost of care for patients hospitalized specifically for hyponatremia was based on the average costs for hospitalizations with a principal discharge diagnosis of hyponatremia (ICD-9-CM code 276.1) as determined from the U.S. Government's Healthcare Cost & Utilization Project's (HCUP) Nationwide Inpatient Sample (NIS) 2002 database. An average cost-to-charge ratio of 0.53, estimated based on publicly available Medicare cost report data [25], was applied to the total billed charges available in the HCUP; and costs were updated to year 2004 U.S. dollars based on the Consumer Price Index for hospital inpatient services[26] A daily ("per diem") cost obtained from a private hospital discharge database was applied to the incremental days in the hospital due to hyponatremia for patients with other conditions. Physician fees associated with office visits, tests, and procedures were based on national prevailing fees for 2004.[27] Facility fees were based on Medicare's Ambulatory Payment Classification (APC) System[28] Unit costs are provided in Table 4.

**Table 4:** Diagnostic tests and procedures

	**Chest X-Ray**	**Basic Metabolic Panel**	**TSH**	**Urine Osmolality**	**ACTH Stimulation Test**	**MRI**	**Chest CT with or without Abdominal Scan**
**Initial Evaluation**							
Patients with CHF, Cirrhosis, Renal Failure, or Taking Diuretics	100%	100%	100%	0%	0%	0%	0%
Patients with SIADH	100%	100%	100%	100%	100%	25%	25%
Patients with All Other Etiologies	100%	100%	100%	100%	100%	0%	0%

**Follow-Up Visits**							

Initial Treatment in Inpatient Setting	100%	100%	0%	0%	0%	0%	0%
Initial Treatment in ER Setting	100%	100%	0%	0%	0%	0%	0%
Initial Treatment in Office Setting	80%–90%	90%	0%	0%	0%	0%	0%

## Results

### Prevalence of hyponatremia in U.S

The prevalence estimates ranged from a low of 3.16 million to a high of 6.07 million persons with hyponatremia in the U.S. on an annual basis. This represents approximately 1.1%–2.1% of the total U.S. population. We found the combination that yielded the lowest prevalence estimate to be the one that used the 'low' classification estimates (prevalence distributed as 1% acute and symptomatic, 4% acute and asymptomatic, 20% chronic and symptomatic, and 75% chronic and asymptomatic), the 'high' percentage treated estimates (100% for acute and symptomatic, 100% for acute and asymptomatic, 85% for chronic and symptomatic, and 20% for chronic and asymptomatic), and the 'high' percentage treated inpatient estimates (75% for acute and symptomatic, 75% for acute and asymptomatic, 45% for chronic and symptomatic, and 80% for chronic and asymptomatic). Using this combination of estimates, we calculated that 23,400 patients are treated for hyponatremia in an inpatient setting for every 100,000 individuals with hyponatremia. Based on the 2002 estimated total of 738,778 patients treated for hyponatremia in an inpatient setting, this places the overall U.S. prevalence at 3.16 million.

The combination that yielded the highest prevalence estimate was the one that used the 'high' classification estimates (prevalence distributed as 1% acute and symptomatic, 4% acute and asymptomatic, 15% chronic and symptomatic, and 80% chronic and asymptomatic), the 'low' percentage treated estimates (90% for acute and symptomatic, 90% for acute and asymptomatic, 66% for chronic and symptomatic, and 10% for chronic and asymptomatic), and the 'low' percentage treated inpatient estimates (65% for acute and symptomatic, 65% for acute and asymptomatic, 40% for chronic and symptomatic, and 70% for chronic and asymptomatic). Based on this combination of estimates, 12,164 patients are treated for hyponatremia in an inpatient setting for every 100,000 individuals with hyponatremia. This places the overall U.S. prevalence at 6.07 million.

### Treatment by setting

Table 2 provides a breakdown of the number and percentage of treated hyponatremia patients who receive initial treatment in each setting of care. Estimates are provided for both the low and high prevalence scenarios. The expert panel agreed that a low percentage of patients with hyponatremia would be treated solely in the office/clinic setting, and that chronic asymptomatic patients would not be seen in the ER. Chronic hyponatremia was estimated to account for greater than 80% of patients initially treated in an inpatient setting, greater than 85% of patients initially treated in an ER, and generally all patients initially treated in an office/clinic setting. Overall, 55%–63% of persons with hyponatremia who are treated are estimated to receive their initial treatment in an inpatient setting, 25% are estimated to be treated initially in the emergency room, and 13%–20% are treated solely in the office setting.

There are an estimated 1 million hospitalizations per year in the U.S. with a principal (accounting for 6.6% of the stays) or secondary discharge diagnosis of hyponatremia. Of all patients with hyponatremia in the inpatient setting, it was estimated that 4%–8% were admitted specifically for hyponatremia and 58%–67% required a longer length of stay due to symptomatic hyponatremia, depending upon the low or high prevalence scenario. The estimate of the total number of additional days of hospitalization due to hyponatremia as a comorbid condition ranged from 497,000 to 4.5 million days per year.

### Cost of illness

The direct costs of treating hyponatremia in the U.S. on an annual basis were estimated to range between $1.6 billion (based on the low prevalence scenario) and $3.6 billion (using the high prevalence scenario) (Table 5). Hospitalization costs (including readmissions) accounted for approximately 70% of the total cost of illness. For the 738,778 patients treated in an inpatient setting, hospitalization costs were estimated at $1.1 billion, or $1,528 per patient (low prevalence scenario), to $2.5 billion, or $3,441 per patient (high prevalence scenario). Hospitalization costs for the subset of patients who were admitted not specifically for hyponatremia but for another reason were based strictly on the days their hospital stay was extended due to their hyponatremia. Therefore, the estimated costs for these patients can be attributed solely to the hyponatremia and are independent of any underlying comorbid condition that was their primary reason for admission. Admissions specifically for hyponatremia accounted for approximately 20% of the hospitalization costs, with the remaining 80% attributable to patients admitted for another condition but whose length of stay was extended due to hyponatremia. Follow-up treatment was the second largest cost driver, accounting for 15%–20% of total costs, depending upon the prevalence scenario. In the low prevalence scenario, 577,131 patients were estimated to require follow-up treatment at a cost of $263 million, or $456 per patient. In the high prevalence scenario, the cost for the 754,861 patients requiring follow-up treatment was estimated at $693 million, or $918 per patient.

**Table 5:** Per patient and total costs of care by treatment setting

	**Total # of patients**	**$ per patient**	**Total costs**
**Low Prevalence Scenario**			

Initial treatment in inpatient setting	738,778	$1,069	$789,604,595
Initial treatment in ER	177,063	$587	$103,950,219
Initial treatment in office setting	148,279	$1,049	$155,513,136
Follow-up treatment	473,097	$453	$214,332,436

**Total**			**$ 1,263,400,385**

**High Prevalence Scenario**			

Initial treatment in inpatient setting	738,778	$2,721	$2,009,973,806
Initial treatment in ER	234,749	$381	$89,464,969
Initial treatment in office setting	257,305	$1,057	$271,952,427
Follow-up treatment	639,810	$916	$585,770,794
**Total**			**$ 2,957,161,995**

## Discussion

This study indicates that hyponatremia represents a substantial medical and economic burden in the U.S. There are approximately 1 million hospitalizations per year in the U.S. with a principal or secondary discharge diagnosis of hyponatremia, as well as an estimated 105,000 to 120,000 annual ER visits, and 1.4 million to 3.4 million annual office visits for hyponatremia. The cost of illness estimate of $1.6 billion to $3.6 billion for hyponatremia can be put into perspective by reviewing published direct cost estimates for other conditions (updated to Year 2004 US $), including $788 million for treating children with respiratory syncytial virus [29], $1.5 billion for treating refractory epilepsy in adults [18], $2.3 billion for treating hay fever [30], $23.4 billion for treating urinary incontinence [31], and $23.7 billion for congestive heart failure [32].

There have been no previously published estimates of the total direct costs of treating hyponatremia, but several previous studies corroborate the conclusions of our analysis. Results from a prospective study of 435 patients admitted to a university hospital with evidence of congestive heart failure showed that hyponatremia (defined as serum [Na^+^] less than or equal to 135 mEq/L) was significantly (*P *≤ 0.01) and independently associated with an increased duration of hospital stay and higher hospital cost [10]. The increased length of hospital stay in patients with hyponatremia was demonstrated in another retrospective analysis of 1,046 patients (58% older than 65 years) hospitalized for heart failure [13]. In this study, 171 patients had hyponatremia (defined as serum [Na^+^] less than 135 mEq/L) at admission and their mean length of stay was 5.78 days, versus 4.72 days among patients without hyponatremia (*P *= 0.0001). The only variable other than hyponatremia that was associated with a longer duration of hospitalization in this study was admission from a skilled nursing facility (6.22 days). A multivariate linear regression analysis indicated that hyponatremia was a significant predictor of hospitalization duration in this cohort of patients.

The current study's cost of illness estimate for hyponatremia is most likely a conservative one. The prevalence estimate was based in large part on the number of hospitalizations for hyponatremia as recorded (by ICD-9-CM diagnosis code) in a national database, but there is evidence that the ICD-9-CM code for hyponatremia represents only one-third of the patients admitted to the hospital and experiencing hyponatremia, due to the low sensitivity (30%) of the diagnosis code [33]. In addition, a high proportion of hyponatremia in the hospital setting is iatrogenic [12,34] and hospitals may be reluctant to include the code in the discharge data.

More definitive resource use and cost data from longitudinal, patient-level databases would have been preferred. However, as noted above, existing databases have their own inherent weaknesses due to the lack of sensitivity with the ICD-9-CM diagnosis code for hyponatremia. Future studies should therefore consider a broader national survey of treatment patterns and resource use associated with hyponatremia.

There are additional limitations associated with this analysis. Although previous research has found that consensus panel decisions have a high degree of consistency and validity when compared with clinical practice [20,21] the panel estimates in the current study are uncertain. A variety of formal and informal methods have been developed for use as consensus-building techniques in group decision-making [35]. The consensus development process in this study was a variation of a modified Delphi panel. In the first stage of a two-stage process, participants privately completed a mailed questionnaire. In the second stage, their compiled responses were presented at a face-to-face meeting where the group engaged in open communication to discuss any variations in their responses. The panel members reached consensus as a group on an appropriate estimate for each question, often in the form of a range. Unlike a true Delphi panel where participants never meet directly, a noted strength of the interactive forum is the opportunity the participants have to provide information, insight, and rationales for their responses. However, a limitation of this approach is the potential for decisions to be reached by persuasion rather than consensus due to an influential member of the group. While no single member of the panel in this study appeared to dominate the consensus process, we recognize that social forces such as persuasion and conformity may have influenced panel members' final decisions.

Additional uncertainty in the panel's estimates lay in the subjective nature of their responses. Previous commentaries in the literature have suggested the potential for bias in prevalence estimates provided by practicing clinicians because their experience is based on the duration of illness, severity, and other clinical characteristics of patients who receive treatment [36]. For example, the prevalence of severe and symptomatic hyponatremia may be easier to estimate than the number of patients who have undetectable symptoms. We believe we minimized this potential bias by having a cross-disciplinary panel familiar with the variety of ways hyponatremia can present itself (i.e. acute, chronic, symptomatic, asymptomatic).

Another study limitation is the lack of inclusion of costs associated with complications of hyponatremia, which although rare, can be substantial. The panel felt it would be difficult to quantify the complications for the extremely small percentage of patients who experience these events. Resource use and costs associated with complications vary depending on the nature and severity of the complication. Furthermore, many complications of hyponatremia are neurological with severe long-term sequelae. Therefore, an accurate assessment of the economic burden would have to include direct and indirect costs incurred over time, which would vary depending on several patient, clinical, and treatment factors. Given the high degree of uncertainty associated with estimating the economic impact of complications and the low percentage of patients involved, the panel deemed it most appropriate to exclude complications from the analysis.

The analysis also did not include the indirect costs associated with hyponatremia. The expert panel did not feel qualified to assign levels of work loss or caregiver burden based on the presence of hyponatremia; and there were no data sources available to directly link hyponatremia with work loss. The increased mortality risk that has been linked to hyponatremia [9,10,37] was assumed to apply mostly to non-working elderly populations, and thus the productivity losses due to mortality were considered minimal.

This analysis of the economic impact of hyponatremia raises a number of clinical implications that have not been fully appreciated nor discussed regarding this disorder. The clinical importance of symptomatic hyponatremia has been well appreciated by clinicians over the past decade, both as a result of the morbidity and mortality associated with hyponatremic encephalopathy, as well as that associated from the production of pontine and extrapontine myelinolysis from overly rapid correction of severe hyponatremia [38]. However, both of these situations are relatively rare in terms of overall incidence, likely representing 1% or less of all hyponatremic patients (Table 1). While these dramatic cases have appropriately received much attention in the medical literature, they represent only a small fraction of the resource utilization and costs associated with hyponatremia. Rather, the bulk of the costs attributable to hyponatremia appear to result from a combination of inpatient hospitalization costs (70%) and subsequent follow-up evaluation and treatment (15%–20%), and 80% of these are attributable to those patients for whom hyponatremia was not the primary diagnosis. Thus, these relatively conservative estimates suggest that more than two-thirds of the cost of hyponatremia occurs from patients hospitalized for other conditions whose length of hospital stay is then extended due to coincident hyponatremia. Further analysis of the reasons underlying this observation is therefore indicated.

Several possibilities can potentially explain this association. First, hyponatremia may be a marker of the severity of the underlying disease, in which case hospitalizations are longer simply because the hyponatremic patients represent a sicker cohort of all those with the underlying disorder. Second, hyponatremia may add its own complications to those of the underlying disorder, thereby acting as an independent factor that extends the length of hospital stay due to the intrinsic complications of this disorder. Third, the presence of hyponatremia may limit or otherwise compromise optimal treatment of the underlying disorder. Finally, because newly-discovered hyponatremia represents a metabolic abnormality of uncertain etiology and significance, the medical evaluation required to ascertain the underlying cause of the hyponatremia will necessarily involve investment of additional time and resources. Each of these possible explanations will be considered in greater detail.

Hyponatremia has long been known to occur in association with a variety of underlying conditions, from tumors that synthesize and excrete arginine vasopressin ectopically [39] to disorders such as congestive heart failure and cirrhosis where arginine vasopressin secretion from the posterior pituitary is stimulated by decreased effective circulating blood volume [40]. It is striking that mortality rates have been found to be significantly higher in hyponatremic patients across a broad range of primary disorders, including congestive heart failure and acute myocardial infarctions [41], pulmonary tuberculosis [42], and childhood diarrhea [43]. Perhaps the strongest data for hyponatremia as a marker of disease severity comes from multiple studies of patients with congestive heart failure, which have clearly shown that hyponatremia represents an independent risk factor in patients with heart failure [8], nearly doubling the risk of mortality in this group [44,45]. Most evidence suggests that this association reflects the underlying pathophysiology of the heart failure (i.e., that hyponatremia is a marker of severity of the underlying disease). This is partly based upon the findings that arginine vasopressin is one of the hormones stimulated during the activation of multiple neurohumoral systems that occurs in association with progression of the heart failure. In the SOLVD (Studies of Left Ventricular Dysfunction), subjects with left ventricular dysfunction had significantly higher plasma arginine vasopressin levels compared to controls, and arginine vasopressin levels were highest in the subjects with overt heart failure [46]. While these data support the possibility that case hospitalizations are longer in hyponatremic patients because they represent a sicker cohort of all patients with the underlying disorder, there are a number of reasons to suggest that the elevated plasma arginine vasopressin levels associated with hyponatremia may in fact aggravate disease progression in patients with heart failure. Specifically, the excess water retention caused by arginine vasopressin may cause worsening of congestive heart failure due to diastolic wall stress from the intravascular volume expansion that is caused by the excess retained water; in addition, the elevated arginine vasopressin levels may lead to increased systolic wall stress as a result of arteriolar vasoconstriction produced by activation of vasopressin V1a receptors in the vasculature, and potential stimulation of myocardial hypertrophy because of growth-stimulating effects of vasopressin V1a receptors in the heart. Thus, the assumption that hyponatremia due to increased arginine vasopressin levels is simply a marker of the severity of the underlying left ventricular dysfunction in patients with congestive heart failure rather than a causal factor in the increased mortality of this subgroup has never been directly tested and remains a presumption.

Regardless of whether elevated arginine vasopressin levels and hyponatremia directly contribute to the morbidity and mortality of underlying primary diseases, there is little question that the presence of hyponatremia can and often does interfere with the treatment of underlying diseases through multiple mechanisms. Perhaps most importantly, standard therapy for euvolemic and hypervolemic patients with hyponatremia is fluid restriction in order to prevent further water retention and worsening of the hyponatremia. This necessity can limit therapies that involve concomitant fluid administration to patients, including antibiotic therapy, chemotherapy, and parenteral nutrition. Furthermore, hyponatremic patients with edema-forming diseases such as congestive heart failure and cirrhosis who require aggressive diuresis of retained water and sodium sometimes do not receive as large a dose of diuretics as otherwise might be given because of fears of worsening hyponatremia as a result of the natriuresis produced by conventional diuretic agents. In each case, this would result in prolonging the period to reach the medical endpoint of the hospitalization.

Finally, even if none of the above scenarios apply to a specific case, the current standard of care for newly diagnosed hyponatremia is to ascertain the etiology of the hyponatremia before ascribing it to the underlying disease [47]. This requires a combination of both laboratory and radiological testing (Table 3) that can add several days to hospitalization, or alternatively, the employment of these resources during follow-up visits. In many cases underlying etiologies are not found,[48] raising questions about the efficacy of the minimum diagnostic evaluation that is appropriate for all cases of hyponatremia.

While no study to date has definitively ascertained among the various possible reasons that account for the increased length of stay in patients with coincident hyponatremia, it seems likely that all of the factors postulated as potential causes of increased resource utilization contribute to this occurrence to varying degrees in individual cases.

## Conclusion

In conclusion, approximately 70% of the estimated $1.6 billion to $3.6 billion cost of illness for hyponatremia is attributable to costs incurred in an inpatient setting. The majority of these costs are attributable to the incremental resource utilization for patients who were not admitted specifically for hyponatremia, but whose hospitalization was prolonged due to hyponatremia. While the potential causes for this are multiple and difficult to ascertain with any degree of certainty, it seems likely that newer therapies that may reduce the incidence and severity of hyponatremia in the inpatient setting could minimize the costs of this important clinical disorder.

## Abbreviations

Abbreviation Description

APC Ambulatory Payment Classification System

ER emergency room

HCUP Healthcare Cost & Utilization Project

Na^+ ^serum sodium concentration

NIS Nationwide Inpatient Sample

SIADH syndrome of inappropriate antidiuretic hormone secretion

SOLVD Studies of Left Ventricular Dysfunction

## Competing interests

This study was funded by Yamanouchi Pharma America, Inc., Paramus, New Jersey.

## Authors' contributions

Audra Boscoe and Clark Paramore contributed to the conceptual design, collected data, conducted the analyses, and drafted the manuscript. Joseph Verbalis contributed to the conceptual design and provided critical revision of the manuscript for important intellectual content.
